# Mediating role of muscle quality in the liver–brain axis: integrated analysis of CT markers of body composition, brain aging, and biomarkers

**DOI:** 10.3389/fnagi.2025.1676721

**Published:** 2025-11-17

**Authors:** Minchul Kim, Chongwon Pae, Joon Ho Moon, Inpyeong Hwang, Kyu Sung Choi

**Affiliations:** 1 Department of Radiology, Kangbuk Samsung Hospital, Sungkyunkwan University School of Medicine, Seoul, Republic of Korea; 2 Department of Radiology, Seoul National University Hospital, Seoul, Republic of Korea; 3 Division of Endocrinology and Metabolism, Department of Internal Medicine, Seoul National University Bundang Hospital, Seoul National University College of Medicine, Seongnam, Republic of Korea; 4 Department of Radiology, Seoul National University College of Medicine, Seoul, Republic of Korea

**Keywords:** myosteatosis, liver-brain axis, brain age, hepatic steatosis, opportunistic screening, automatic segmentation

## Abstract

**Background:**

The liver, skeletal muscle, and brain are interconnected through metabolic and endocrine pathways, constituting a systemic axis that may influence neurodegeneration. Although hepatic steatosis and sarcopenia have been independently associated with neurodegeneration, their integrated effects on the brain remain poorly understood. This study investigated whether muscle density mediated the link between hepatic steatosis and neurodegeneration, quantified via the brain age gap (BAG).

**Methods:**

Data from 2,510 adults (aged 22–87 years) who underwent abdominal computed tomography (CT), brain magnetic resonance imaging (MRI), and blood tests during comprehensive health evaluations were retrospectively analyzed. Fully automated CT markers, including visceral and subcutaneous fat, muscle, and the liver attenuation index (LAI) (a CT-based surrogate of hepatic steatosis) were obtained. The BAG was calculated from T1-weighted structural MRI scans using a pretrained machine learning pipeline. Mediation analysis was performed to evaluate the indirect effects of LAI on the BAG through muscle density. Network analysis further characterized the multivariate associations between the BAG, CT markers of body composition, laboratory results, and anthropometric variables.

**Results:**

Mediation analysis confirmed that muscle density, not muscle volume, partially mediated the LAI–BAG relationship (indirect β = −0.04, *p* < 0.001). LAI was negatively associated with the BAG (β = −0.027, *p* = 0.002) and positively associated with muscle density (β = 0.049, *p* < 0.001), whereas muscle density was inversely associated with BAG (β = −0.080, *p* < 0.001). Network analysis identified muscle density as a central hub linking the LAI, body composition, and the BAG. The BAG was also negatively correlated with Montreal Cognitive Assessment scores (*r* = −0.20, *p* < 0.001).

**Conclusion:**

Muscle density mediates the effect of hepatic steatosis on brain aging, supporting its role as a key modifiable factor within the liver–brain axis. These findings underscore the importance of preserving muscle quality to decelerate brain aging.

## Highlights

Recent studies have established the associations between body composition, metabolic dysfunction, and brain aging; however, the complex mechanisms linking multiple metabolic factors to the brain remain underexplored. This study builds on prior work by showing that muscle density, a CT-based marker of myosteatosis, mediates the relationship between hepatic steatosis and brain-predicted age. By integrating multimodal data, including structural brain MRI, abdominal CT-based markers of body composition, laboratory biomarkers, and cognitive performance, our analysis identified muscle quality as a central node within the body-liver-brain axis. The inclusion of inflammatory, glycemic, and hemodynamic markers enables further characterization of systemic biological pathways associated with accelerated brain aging. These findings suggest that skeletal muscle quality may represent a key modifiable factor mediating the relationship between metabolic dysfunction and accelerated brain aging.

## 1 Introduction

The aging of the global population has promoted an increasing prevalence of age-related conditions, such as cognitive decline, metabolic dysfunction, and sarcopenia (Yuan and Larsson, 2023). Despite the progress in life expectancy, no proportional improvement in the healthspan has been noted, highlighting the need to identify modifiable systemic factors that contribute to brain and physical aging (López-Otín et al., 2013; Wrigglesworth et al., 2021). Recent evidence suggests that physical and cognitive health are interdependent, with numerous studies demonstrating a bidirectional relationship between metabolic dysfunction and the brain (Zhao et al., 2021).

The liver is the primary organ involved in maintaining energy balance among humans given that it controls the metabolism of different nutrients, such as lipids, glucose, and protein (Matsubara et al., 2022). Alteration in lipid metabolism leads to the accumulation of lipids in the liver, resulting in organelle malfunction, cellular injury, inflammation, and persistent activation of pathways associated with fibrosis, all of which worsen liver function and promote the development of metabolic dysfunction-associated steatotic liver disease (MASLD) (Rao et al., 2023). Extrahepatic manifestations of MASLD have attracted the attention of researchers in the field of both hepatology and neuroscience. Interest in cognitive functioning and brain health has been particularly high owing to their shared risk factors and pathophysiology with liver diseases (Weinstein et al., 2018). This relationship forms the basis of the emerging liver–brain axis, a conceptual model describing the dynamic interaction between the brain and liver (Matsubara et al., 2022). Specifically, recent efforts to understand the liver–brain axis have identified a link between liver function and imaging markers of brain structure, implying accelerated brain aging (Jiang et al., 2023; Wang et al., 2025).

Disturbed hepatic lipid metabolism affects not only the brain but also the muscles. In fact, MASLD has been closely related to various metabolic complications, such as obesity, insulin resistance, type 2 diabetes, hyperlipidemia, hypertension, and other cardiovascular diseases, due to the significant role hepatic lipid metabolism plays in the overall energy balance of the body (Rao et al., 2023). The Framingham Heart Study found that lower computed tomography (CT) attenuation of the paraspinal muscle, which was a marker of myosteatosis and therefore muscle quality, was associated with various metabolic risk factors, such as hyperglycemia, dyslipidemia, and hypertension (Kim J. A. et al., 2025; Therkelsen et al., 2013). Interestingly, recent evidence suggests that muscle health and metabolic indices, including fasting glucose levels and C-reactive protein levels, are also linked to brain aging (Lee et al., 2025). Hence, disruptions in this multiorgan axis (i.e., hepatic steatosis and myosteatosis) may collectively accelerate brain aging through shared metabolic pathways. Although sarcopenia and hepatic steatosis have been individually associated with brain atrophy, no study has yet clearly established the mediating role of muscle in the association between liver fat accumulation and brain aging.

The brain age gap (BAG), defined as the difference between the predicted brain age based on magnetic resonance imaging (MRI) and chronological age, is a robust marker of neurodegeneration. A higher BAG, which implies accelerated aging, has been associated with cognitive impairment, neuropsychiatric conditions, and metabolic risk factors (Cole et al., 2018; Franke and Gaser, 2019). Previous studies have shown that BAG was clearly associated with hepatic steatosis and myosteatosis, indicating that people with higher measures of adipose tissue have older-appearing brains and that BAG was a reasonable surrogate for brain health (Beck et al., 2022; Lee et al., 2025).

Although some pieces of the puzzle have been investigated, only a few studies have examined the BAG within the context of whole-body metabolic status or organ–organ interactions. Thus, no integrated model with an all-in-one analysis has yet been established. Considering the interdependency among organs in regulating systemic inflammation and glucose and lipid metabolism, we hypothesized that muscle density would mediate the effects of hepatic fat on brain aging, positioning it as a critical intermediary within the body–liver–brain axis. Using a large retrospective cohort of 2,510 adults who underwent abdominal CT, brain MRI, and metabolic profiling, a multimodal analysis that integrated imaging-derived markers, blood biomarkers, and anthropometric data was conducted. Specifically, we employed mediation analysis to determine whether myosteatosis mediates the association between hepatic steatosis and BAG. Additionally, graphical least absolute shrinkage and selection operator (LASSO)-based network analysis was used to elucidate dependencies across multiple variables.

## 2 Materials and methods

### 2.1 Patient selection and demographics

This retrospective study was approved by the Institutional Review Board of Seoul National University Hospital (SNUH IRB No. 2504-076-1629) and conducted in accordance with the Declaration of Helsinki. Informed consent was waived due to retrospective nature of the study. We examined data from 2510 consecutive individuals (1294 males, 1216 females) ranging in age from 22 to 87 years. These participants had completed a comprehensive health check-up program at the Seoul National University Hospital Health Promotion Center in the Republic of Korea, with data collected between January 2019 and December 2022. The health assessment battery for each participant involved a combination of laboratory analyses, anthropometric measurements, a questionnaire about smoking history, abdominal computed tomography, and brain MRI. For laboratory analyses, venous blood samples were collected before 10:00 AM after a 12-h fast. Standardized methodologies were employed for all biochemical analyses, which were conducted at a single laboratory (Lee et al., 2010). A small portion of the included patients (220 participants) underwent the Korean version of the Montreal Cognitive Assessment (MOCA) (Kang et al., 2009).

### 2.2 MRI, quality control, and brain age estimation

Magnetic resonance imaging of the brain was performed using a GE Discovery MR750w 3.0-T system (GE Healthcare, Milwaukee, WI), equipped with a 24-channel head coil. The T1-weighted images were captured via a three-dimensional (3D) fast spoiled gradient-echo sequence. Key acquisition parameters were: 8.0 ms repetition time, 3.0 ms echo time, 450 ms inversion time, and a 12° flip angle. The images had a 256 mm × 256 mm field of view, a 256 × 256 acquisition matrix, and a 139.4 Hz/pixel receiver bandwidth, with 1 excitation. Sagittal slices, varying between 154 and 172 based on head size, were acquired at a 1 mm thickness, yielding a 1 mm^3^ voxel resolution. Consistent with previous research, high-quality scans were defined by the absence of imaging artifacts (such as ghosting or ringing), no evidence of prior brain pathology (e.g., lacunar infarctions), and an Euler number (rescaled) below 10, indicating good scan integrity (Kim M. et al., 2025). Brain age was calculated using brainageR (version 2.1), an open-access software for generating brain age predictions from raw T1-weighted MRI scans^1^ (Cole et al., 2018). BrainageR involves two main stages: preprocessing and prediction. In the preprocessing stage, images are segmented and normalized via SPM12 software^2^. For quality control, the FSL slicesdir function was used to generate two-dimensional slices of the segmentation and normalization outputs. Normalized images were loaded into R (R Core Team, 2013) and converted to vectors. Gray matter, white matter, and cerebrospinal fluid vectors were masked using a 0.3 threshold from the mean image template based on the brainageR model training dataset and then combined. In the prediction stage, the brainageR model was applied to the vectorized and masked study images to estimate a brain age score for each. BrainageR had been previously trained to predict the brain age from normalized brain volumetric maps obtained from 3377 healthy individuals included in seven publicly available datasets using the Gaussian Processes Regression. Using principal component analysis, the top principal components capturing 80% of the variance in brain volumes were retained. The resulting rotation matrix for the 435 principal components was then applied to the new imaging data to predict the brain age (Biondo et al., 2022).

For each image, the final output of brainageR was a predicted brain age value with the corresponding 95% confidence interval (CI). After calculating the predicted brain age for each subject, we further calculated the BAG, a metric that reflected the subject’s relative brain health status. The BAG was initially measured by subtracting the true brain age from the predicted brain age, with higher values implying an older brain morphology relative to the participant’s chronological age. Owing to regression dilution, however, regression models may also bias the predicted brain age toward the mean, which can underestimate the age of older subjects and overestimate the age of younger participants. To correct this bias, we defined the BAG as the difference between the individual BAG and the expected BAG (measurements fitted over the entire sample set using the regression model and leave-one-out cross-validation) (Kang et al., 2023). The BAG was then corrected such that the BAGs of the whole dataset analyzed became unbiased across all age ranges.

### 2.3 Acquisition of abdominal CT images

A dual-source scanner (SOMATOM Force; Siemens Healthineers, Erlangen, Germany) was used for all computed tomography procedures. After a bolus injection of iobitridol (Xenetix 350; Guerbet) at 520 mg/kg body weight, followed by a saline flush, images were acquired during the single portal venous phase 70 s after contrast media delivery. Data collection occurred in dual energy mode, utilizing 80 and 150 kVp settings. Virtual non-contrast images were then derived from the dual energy data using a dedicated Syngo.via post-processing system (Siemens Healthineers) directly at the CT console, and reconstructed with a 2-mm slice thickness (Jeon et al., 2024).

Volumetric quantification of the liver and spleen was performed automatically using DeepCatch version 1.2.0.0 (MEDICALIP Co., Ltd., Seoul, Korea), a deep-learning-based multiorgan segmentation software, applied to the virtual non-contrast enhanced images. The software’s reported segmentation performance for these organs, on both non-contrast and post-contrast enhanced images, demonstrated Dice scores greater than 0.95 (Jeon et al., 2024). Following the creation of a 3D organ mask, the system automatically computed the mean volumetric CT attenuation (in Hounsfield units, HU) for the segmented regions.

### 2.4 Variable selection and statistical analysis

For integrative analysis of the brain–liver–muscle axis, we selected body composition measures and serum biomarkers that were useful, or may be potential systemic confounders in evaluating metabolic dysfunction and brain aging based on previous studies. We employed the liver attenuation index (LAI) (mean CT attenuation of the liver - mean CT attenuation of the spleen) as a CT attenuation-based parameter for assessing hepatic steatosis in accordance with previous studies on the utility of CT for this purpose. The LAI has been reported to be robust across various scan settings using the spleen as an internal reference (Jeon et al., 2024). We used skeletal muscle and visceral fat volume (in cm^3^), normalized to height (in meters squared), as indices (termed as MusIndex and VisFatIndex, respectively) (Chang et al., 2024). This study focused on these volumes, as well as the visceral-to-subcutaneous fat ratio (AVF_SFVolumeRatio), given their strong predictive value for overall mortality and association with type 2 diabetes mellitus (Chang et al., 2024). We also selected the mean CT attenuation of the muscle as the gold standard for evaluating myosteatosis (Kim J. A. et al., 2025) given its association with metabolic risk factors, such as hyperglycaemia, dyslipidaemia, and hypertension (Therkelsen et al., 2013). Body-mass index (BMI) was included given its potential to be an independent risk factor for diffuse brain alterations manifesting as accelerated brain age (Kolenic et al., 2018). Among the laboratory tests, we included hemoglobin A1c (HbA1c), triglycerides (TG), and high-sensitivity C-reactive protein (hs-CRP) to represent hyperglycemia, dyslipidemia, and systemic inflammation (Wang et al., 2025). Lastly, mean arterial pressure (MAP) was included as the single marker of blood pressure (Kulshreshtha and Chandel, 2025). All conditions were found to be linked to brain health or cognition (Jawinski et al., 2022).

**FIGURE 1:**
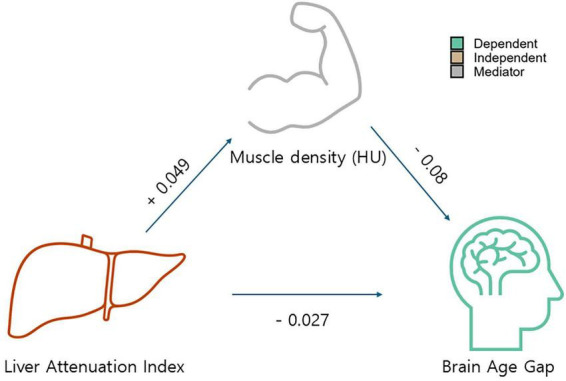
Model showing the mediating effects of muscle density (Hounsfield unit) on the ability of the liver attenuation index as a predictor of accelerated brain aging. The direction of the arrows, as opposed to the coefficients, is based on rationale supported by previous literature; however, we acknowledge that this represents a suggestive rather than definitive framework.

We investigated the association between imaging-derived markers, blood biomarkers, and anthropometric data with BAG. First, a mediation model (Figure 1) was built to examine how the independent variable (LAI) affected the dependent variable (BAG) through an intermediary variable known as the mediator (mean CT attenuation of the muscle as a marker of myosteatosis). Mediation analysis decomposes the total exposure (X, LAI)-outcome (Y, BAG) effect into a direct effect and an indirect effect through a mediator (M, myosteaosis); therefore, it is an important statistical tool for gaining insight into the mechanisms of exposure-outcome effects (MacKinnon, 2012). This type of analysis allows us to understand the mechanisms behind the observed effects, which can lead to more targeted interventions. We evaluated muscle density as a mediator of *de novo* lipogenesis considering that systemic inflammation and metabolic disturbances can leading to increased fat infiltration into the muscle and that both hepatic steatosis and myosteaosis has been linked to cognitive decline and accelerated brain aging (Altajar and Baffy, 2020). To support the role of myosteatosis as a mediator and our data is cross-sectional, we conducted same mediation analysis with exposure and outcome reversed. The PROCESS macro for R statistical programming language developed by Hayes (2017) was used to compute the models (R Core Team, 2013). We additionally tested a mediation model with the MusIndex as a mediator for sensitivity analysis.

Second, network analysis was applied to characterize the relationships among all selected variables using the JASP network analysis module (Costantini et al., 2015; Smarandache et al., 2022; JASP Team, 2025). The network model included participants’ chronological age, given the wide age range in our dataset, and was stratified by sex to control for potential imbalances between sexes. During network analysis, variables are referred to as nodes, and relationships among nodes are referred to as edges. The strength of the relationship between the nodes is indicated in terms of edge weights. The graphical LASSO procedure simplifies the interpretation of the network by penalizing small correlation values to zero. Given that this replacement serve as a tradeoff between false-positive correlations (i.e., correlations with small values that should be removed) and true-positive correlations (i.e., correlations with small values that should not be removed), we used a version of the LASSO regularization that uses the extended Bayesian information criterion (EBIC) model selection (Foygel and Drton, 2010) with the default hyperparameter gamma (γ). We also computed the 95% CIs for the edges of the EBIC-regularized LASSO network using a non-parametric bootstrap procedure with 1000 randomly selected samples.

Lastly, we explored the relationship between MOCA scores and the BAG and muscle density in the subgroup of participants with available MOCA scores.

## 3 Results

The clinical characteristics and descriptive statistics of the study population are summarized in Table 1. Figure 1 and Table 2 present the results regarding the mediating effect of muscle density (i.e., a proxy for myosteatosis and thus muscle quality) on ability of the LAI to predict the BAG. Regression analysis revealed that muscle density had a significant negative effect on the BAG (β = −0.0795, SE = 0.0205, *t* = −3.8785, *p* < 0.001), indicating that a decrease in muscle quality was associated with accelerated brain aging. We also found that LAI had a significant positive effect on muscle density (β = 0.0489, SE = 0.0084, *t* = 5.8575, *p* < 0.001), suggesting that higher liver adiposity is associated with lower muscle quality. The direct effect of the LAI on the BAG, controlling muscle density, was also significant (β = −0.0271, SE = 0.0086, *t* = −3.1392, *p* = 0.0017), reconfirming the direct influence of liver adiposity on brain aging. Lastly, our results showed that the bootstrapped indirect effect, which is the proportion mediated by muscle quality, was significant (effect = −0.0039, bootstrapped SE = 0.0012, 95% CI −0.0065, −0.0017]) given that the confidence interval did not include zero. This confirmed that the decrease in muscle quality related to hepatic steatosis partially mediates liver-related brain aging, explaining approximately 8% of the total effect. The sensitivity mediation analysis with revered directional hypothesis also revealed that muscle density as a mediator (Supplementary Figure 1). In another sensitivity analysis (Supplementary Figure 2) wherein muscle density was replaced with the MusIndex (the normalized muscle volume), we found that MusIndex was positively associated with the BAG, suggesting that increased muscle volume was associated with accelerated brain aging. This finding contradicts common knowledge, which implies a confounder between MusIndex and the BAG (e.g., increased BMI inflates low-quality muscle volume).

**TABLE 1:** Clinical characteristics of the study population.

Variables	Men *n* = 1,294	Women *n* = 1,216
Age (years)	60.59 (9.226)	61.143 (8.609)
Predicted brain age (years)	52.672 (11.702)	50.812 (11.423)
Absolute brain age gap (years)	5.864 (4.399)	5.837 (4.458)
LAI	−20.67 (17.899)	−16.349 (15.655)
MAP	91.716 (11.634)	89.518 (12.198)
BMI	24.927 (3.165)	23.442 (3.315)
VisFatIndex	0.125 (0.056)	0.087 (0.049)
MusIndex	0.285 (0.040)	0.227 (0.030)
AVF_SFVolumeRatio	1.28 (0.66)	0.514 (0.357)
HbA1c	5.954 (0.839)	5.848 (0.747)
hsCRP	0.196 (0.612)	0.172 (0.572)
TG	121.759 (80.19)	95.264 (53.057)
History of over 1 pack-year smoking	831	56
MOCA (*n* = 220)	25.565 (3.361) (*n* = 85)	24.393 (4.123) (*n* = 135)

Continuous data are means (SD). LAI, liver attenuation index; MAP, mean arterial pressure; BMI, body-mass index; VisFatIndex, visceral fat volume index; MusIndex, muscle volume index; AVF_SFVolumeRatio, visceral-to-subcutaneous fat volume ratio; HbA1c, glycated hemoglobin; hsCRP, high-sensitive C-reactive protein; TG, triglycerides; MOCA, Korean version of the Montreal Cognitive Assessment.

**TABLE 2:** Mediation analysis results for the direct, indirect, and total effects, with liver attenuation index (LAI) as the independent variable, muscle density (HU) as the mediator, and the brain age gap (BAG) as the dependent variable.

Effect type	Pathway	Effect (B)	SE	t	*p*	95% CI (lower)	95% CI (upper)
Direct effect	LAI → BAG (c’)	−0.0271	0.0086	−3.1392	0.0017	−0.0441	−0.0102
Indirect effect	LAI → Muscle density → BAG (a × b)	−0.0039	0.0012	–	–	−0.0065	−0.0017
Total effect	LAI → BAG (c)	−0.031	0.0087	−3.5643	0.0004	−0.048	−0.014

LAI, liver attenuation index; BAG, brain age gap; SE, standard error; CI, confidence interval.

**FIGURE 2:**
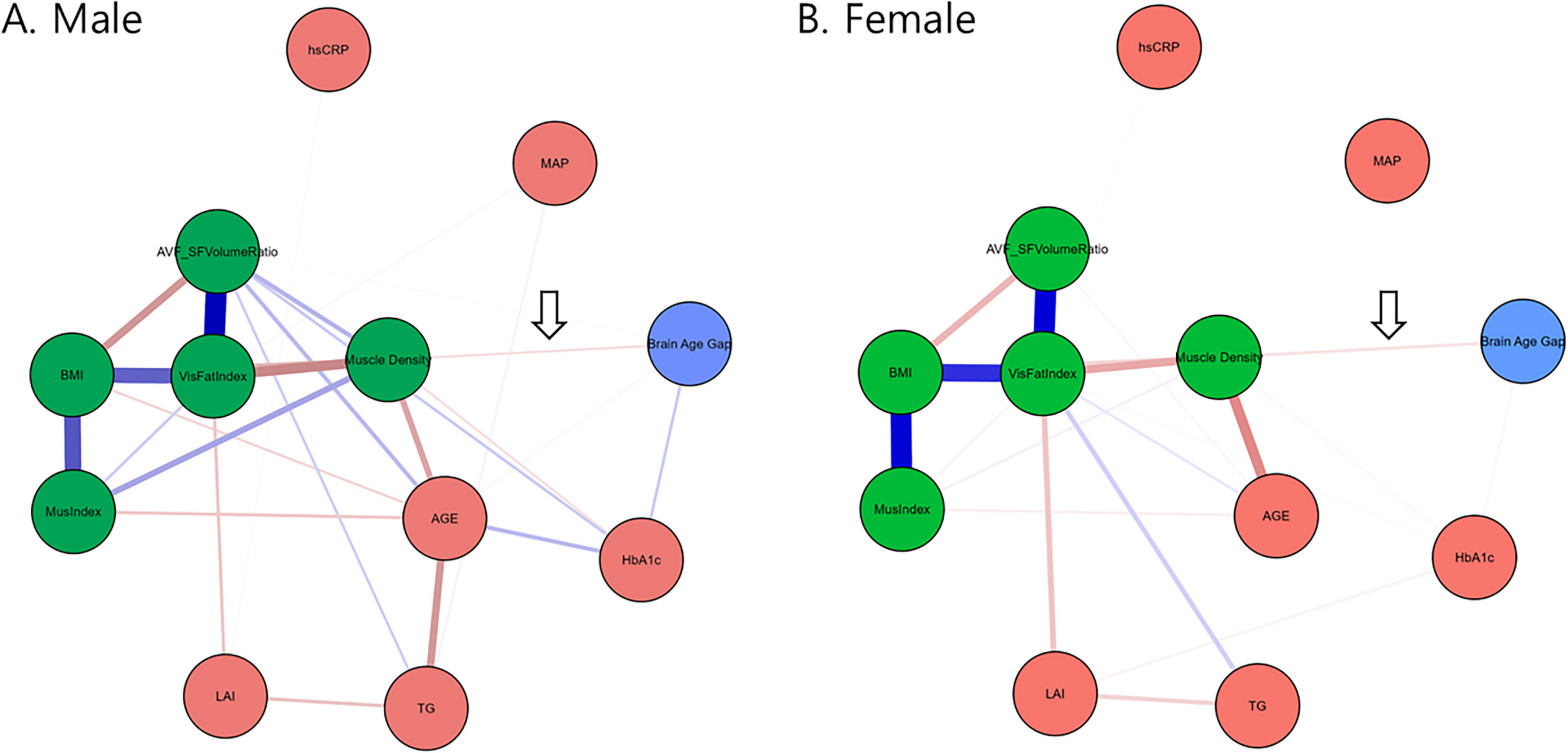
Networks constructed via graphical LASSO depicting regularized partial correlations among variables from the BAG, CT-derived markers, laboratory test measures, and anthropometric measurements stratified according to sex. Nodes representing CT-derived muscle and fat markers are colored green. The arrow indicates that muscle density exclusively connects the green nodes to the BAG. The blue lines represent positive associations, whereas the red lines represent negative associations. The thickness and brightness of the edges indicate the strength of the associations. LAI, liver attenuation index; MAP, mean arterial pressure; BMI, body-mass index; VisFatIndex, visceral fat volume index; MusIndex, muscle volume index; AVF_SFVolumeRatio, visceral-to-subcutaneous fat volume ratio; HbA1c, glycated hemoglobin; hsCRP, high-sensitive C-reactive protein; TG, triglycerides; MOCA, Korean version of the Montreal Cognitive Assessment, AGE, subject’s chronological age.

Next, we applied network analysis to characterize the relationships among all selected variables that may confound the complex relationship. The graphical LASSO network analysis (Figure 2) also suggested that muscle density serves as a critical link between body composition and brain aging (arrows in Figure 2). In both male and female networks, the BAG node (blue) connects exclusively to the four CT-derived body composition measures (green nodes) through muscle density. Additionally, our results showed that the LAI was linked to visceral fat volume and serum markers of HbA1c (notably in men, Figure 2A) and TG but showed no direct connection to the BAG, implying that systematic metabolic alterations caused by hepatic steatosis affect muscle density and consequently promote brain aging. The bootstrapped confidence intervals of all edge weights in the two networks are detailed in Supplementary Figure 3.

**FIGURE 3:**
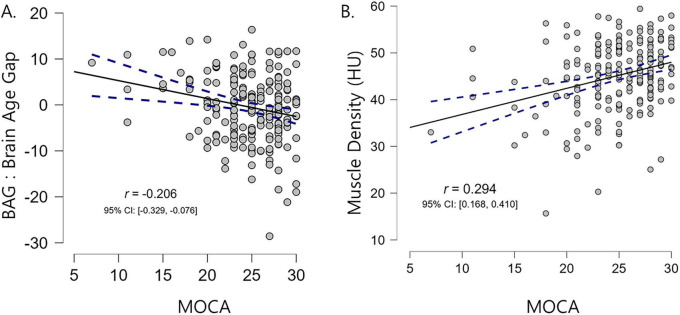
Correlation scatter plots illustrating the relationships between MOCA scores (*x*-axis) and the brain age gap **(A)** and muscle density **(B)**. Higher MOCA scores were associated with lower brain age gaps and higher muscle density values. Pearson’s correlation coefficients (*r*) and 95% confidence intervals are also reported.

We also examined the correlations between the BAG and MOCA scores, as well as between muscle density and MOCA scores. Notably, we found a significant negative correlation between the BAG and MOCA scores (*r* = −0.20, *p* < 0.001) but a significant positive correlation between muscle density and MOCA scores (*r* = 0.29, *p* < 0.001) (Figure 3). These findings align with those reported in previous research demonstrating association between brain aging, myosteatosis, and cognitive function (Liem et al., 2017).

## 4 Discussion

The current study found that the muscle quality index (specifically, muscle density) mediates the relationship between hepatic steatosis measured using the LAI and accelerated brain aging measured using the BAG (Figure 1 and Table 2). Furthermore, by integrating widely used markers of body composition, serum biomarkers, and metabolic indicators into a graphical LASSO network, we observed that body composition and the LAI were associated with BAG exclusively through muscle density (Figure 2, arrows). Given that the network was plotted using the Fruchterman–Reingold algorithm, which places most central nodes into the center (Fruchterman and Reingold, 1991), the central placement of the muscle density within the network underscores its pivotal role among the analyzed variables associated with metabolism. This finding suggests that muscle density plays a crucial intermediary role in the body–brain axis. Additionally, our findings reaffirmed the negative association between brain aging and cognitive function, as well as between muscle density and cognitive function (Figure 3), consistent with previous research indicating a link between increased brain age and cognitive decline (Elliott et al., 2021).

After constructing a hypothetical model based on the previously established liver–brain–muscle axis, we found that myosteatosis additionally contributes to the association between hepatic steatosis and brain aging. However, the directionality of this relationship–whether from liver to brain or vice versa–remains unconfirmed. The directionality of the model represents the authors’ best estimate, informed by a review of the existing literature; the liver and brain engage in a well-established bidirectional dialogue known as the “liver–brain axis” such that pathological conditions in the liver can contribute to the development of neurodegenerative disorders, while neurological states can impact liver function (Nguyen and Swain, 2023). Several large-scale studies linking hepatic steatosis with accelerated brain aging (VanWagner et al., 2017; Weinstein et al., 2018) have confirmed that the brain and liver constantly communicate through hepatokines, metabolites, and autonomic nerves to regulate metabolism (De Cól et al., 2024). Liver steatosis disrupts gut barrier integrity and perturbs bile acid and cytokine homeostasis, allowing endotoxins and inflammatory mediators to cross the blood–brain barrier and trigger neuroinflammation and structural atrophy (Yan et al., 2023). Bile acids provide neuroprotection through a complex system of physiological mechanisms; after being derived from the liver, bile acids are transformed by gut microbiota, and interact with receptors such as the Farnesoid X and G protein-coupled bile acid receptor along the intestinal and brain axis, eventually providing a role in mood regulation, cognition, anti-inflammatory responses, and neuroprotection (Yassin et al., 2025). Furthermore, studies have incorporated systemic metabolic factors to explain the complex crosstalk between the liver and brain (Jiang et al., 2023; Weinstein et al., 2018). Skeletal muscle also communicates with the brain through several known mechanisms. During exercise, skeletal muscle releases myokines, such as brain-derived neurotrophic factor (BDNF), which can cross the blood–brain barrier and exert beneficial effects on brain function, including enhanced learning, memory, and mood (Yin et al., 2022). Irisin, another myokine induced by exercise, also upregulates BDNF, particularly in the hippocampus, and has neuroprotective effects (Jodeiri Farshbaf and Alvina, 2021). As such, emerging evidence has indicated an association between skeletal muscle health and brain aging markers (Samuelsson et al., 2025; Yu et al., 2021). The literature suggests that fatty liver often occurs concurrently with reduced muscle density (myosteatosis) and sarcopenia. Indeed, studies have indicated a possible common mechanism (e.g., insulin resistance and chronic inflammation) linking fatty liver with muscle deterioration (Pasco et al., 2022). Specifically, NAFLD induces systemic insulin resistance, which promotes intramyocellular lipid accumulation and, consequently, myosteatosis through impaired glucose disposal in muscle tissue (Samuel and Shulman, 2012). In addition, myosteatosis is accompanied by chronic low-grade inflammation–driven by increased proinflammatory cytokines and oxidative stress–that further disrupts the muscle–liver–adipose axis and may exacerbate both hepatic and neural injury (Henin et al., 2024). Similarly, a recent study discovered that sarcopenia was strongly and independently associated with a higher risk of mortality in patients with liver cirrhosis (Tantai et al., 2022). Therefore, our findings – muscle density mediates the impact of liver steatosis on brain aging – can be explained by several well-characterized biological pathways.

Our research took advantage of opportunistic screening, which was made possible by the development of deep learning automatic segmentation models (Pickhardt, 2022). Although we did not directly measure muscle strength, large-scale studies have consistently confirmed that myosteatosis quantified through CT attenuation, rather than muscle volume itself, was a critical factor in staying healthy and preventing cardio-metabolic diseases (Chang et al., 2024; Kim J. A. et al., 2025). Kim J. A. et al. (2025) discovered that muscle density was positively associated with greater muscle strength and better physical performance in men and women, regardless of muscle mass or diabetes status. Hence, improving myosteatosis may be a therapeutic target for preventing sarcopenia. Similarly, Chang et al. (2024) reported that patients with type 2 diabetes mellitus exhibited increased muscle mass but decreased muscle density on CT. Identifying low muscle density, which indicates myosteatosis, is crucial given its adverse association with muscle strength and mortality (Nachit et al., 2023). Our results further highlight muscle density as an important link between cardio-metabolic conditions and neurodegeneration. Given that regular exercise can enhance muscle quality, particularly in healthy populations, regular physical activity should be recommended more often (Kim J. A. et al., 2025).

Although our study identified significant associations between the LAI, muscle density, and the BAG, the observed effect sizes were quite modest. This indicates that while LAI and muscle density contributed to variations in brain aging, they accounted for a limited portion of the variance, with a total effect of β = −0.0310. Consequently, other unmeasured factors may have a more substantial impact on brain age. For example, the microbiota–gut–brain axis is increasingly recognized as a critical regulator of brain health, influencing both neurodevelopment and age-related neurological decline. Unlike the brain, the gut microbiota is directly accessible to external influences, including dietary changes, prebiotics, probiotics, antibiotics, and other lifestyle-related interventions (Yassin et al., 2025). Future research should aim to identify these additional variables to provide a more comprehensive understanding of the determinants of brain aging.

The current study has a few limitations worth noting. First, the current research relies on a retrospective and cross-sectional design. Although our model was developed based on previous evidence on hepatic steatosis, myosteatosis, and the BAG, the observed mediation effects are strictly a measure of association and cannot determine causality. Given the potential flaws associated with mediation analysis, these results should be considered preliminary (Zhao et al., 2010). A future study with a prospective, repeated-measures design may strengthen our findings and establish causality. Second, we observed a relatively large BAG (Supplementary Figure 4), which may be attributed to the advanced average age of our participants (approximately 60 years old) and potential ethnic differences compared to the training dataset. However, the mean absolute error (MAE) of approximately 5 years in our study aligns with previous research conducted on a large East Asian population. For instance, a recent study by Lee et al. (2025) reported an MAE of 4.26 and 6.11 years in their training and clinical test dataset, respectively. Lastly, the precise molecular mediators that link myosteatosis to region-specific cortical thinning are undetermined, and the relative contributions of different myokines require elucidation. Moreover, while the liver–brain axis is recognized, the specific metabolites or cytokines mediating direct hepatic effects on neural structure remain to be identified. Addressing these gaps will demand targeted mechanistic studies integrating lipidomics, cytokine profiling, and regionally resolved neuroimaging. Finally, there is a need for clinical trials to prove whether improving skeletal muscle quality can serve as a modifiable therapeutic target to preserve brain health in hepatic steatosis.

In summary, the current study involving a large cohort of participants provides an integrative model highlighting the role of muscle quality in accelerated brain aging and cognitive functioning. Considering that myosteatosis can serve mediate the relationship between body composition and neurodegeneration, improving myosteatosis may be the key modifiable factor within the liver–muscle–brain axis.

## Data Availability

The raw data supporting the conclusions of this article will be made available by the authors, without undue reservation.

## Footnotes

1
https://github.com/james-cole/brainageR

2
https://www.fil.ion.ucl.ac.uk/spm/software/spm12/

## References

[B1] Altajar S. Baffy G. (2020). Skeletal muscle dysfunction in the development and progression of nonalcoholic fatty liver disease. *J. Clin. Transl. Hepatol.* 8 414–423. 10.14218/JCTH.2020.00065 33447525 PMC7782111

[B2] Beck D. de Lange A. G. Alnæs D. Maximov I. I. Pedersen M. L. Leinhard O. D. (2022). Adipose tissue distribution from body MRI is associated with cross-sectional and longitudinal brain age in adults. *Neuroimage Clin.* 33:102949. 10.1016/j.nicl.2022.102949 35114636 PMC8814666

[B3] Biondo F. Jewell A. Pritchard M. Aarsland D. Steves C. J. Mueller C. (2022). Brain-age is associated with progression to dementia in memory clinic patients. *Neuroimage Clin.* 36:103175. 10.1016/j.nicl.2022.103175 36087560 PMC9467894

[B4] Chang Y. Yoon S. H. Kwon R. Kang J. Kim Y. H. Kim J. M. (2024). Automated comprehensive CT assessment of the risk of diabetes and associated cardiometabolic conditions. *Radiology* 312:e233410. 10.1148/radiol.233410 39105639

[B5] Cole J. H. Ritchie S. J. Bastin M. E. Hernández V. Muñoz Maniega S. Royle N. (2018). Brain age predicts mortality. *Mol. Psychiatry* 23 1385–1392. 10.1038/mp.2017.62 28439103 PMC5984097

[B6] Costantini G. Epskamp S. Borsboom D. Perugini M. Mõttus R. Waldorp L. J. (2015). State of the aRt personality research: A tutorial on network analysis of personality data in R. *J. Res. Personal.* 54 13–29. 10.1016/j.jrp.2014.07.003

[B7] De Cól J. P. de Lima E. P. Pompeu F. M. Cressoni Araújo A. de Alvares Goulart R. Bechara M. D. (2024). Underlying mechanisms behind the brain-gut-liver axis and metabolic-associated fatty liver disease (MAFLD): An update. *Int. J. Mol. Sci.* 25:3694. 10.3390/ijms25073694 38612504 PMC11011299

[B8] Elliott M. L. Belsky D. W. Knodt A. R. Ireland D. Melzer T. R. Poulton R. (2021). Brain-age in midlife is associated with accelerated biological aging and cognitive decline in a longitudinal birth cohort. *Mol. Psychiatry* 26 3829–3838. 10.1038/s41380-019-0626-7 31822815 PMC7282987

[B9] Foygel R. Drton M. (2010). “Extended Bayesian information criteria for Gaussian graphical models,” in *Proceedings of the 24th International Conference on Neural Information Processing Systems*, (New York, NY: ACM).

[B10] Franke K. Gaser C. (2019). Ten years of *BrainAGE* as a neuroimaging biomarker of brain aging: What insights have we gained? *Front. Neurol.* 10:789. 10.3389/fneur.2019.00789 31474922 PMC6702897

[B11] Fruchterman T. M. Reingold E. M. (1991). Graph drawing by force-directed placement. *Software: Pract. Exp.* 21 1129–1164. 10.1002/spe.4380211102

[B12] Hayes A. F. (2017). *Introduction to mediation, moderation, and conditional process analysis: A regression-based approach.* New York, NY: Guilford publications.

[B13] Henin G. Loumaye A. Leclercq I. A. Lanthier N. (2024). Myosteatosis: Diagnosis, pathophysiology and consequences in metabolic dysfunction-associated steatotic liver disease. *JHEP Rep.* 6:100963. 10.1016/j.jhepr.2023.100963 38322420 PMC10844870

[B14] JASP Team (2025). *JASP (Version 0.19.3)[Computer software].* Amsterdam: JASP Team.

[B15] Jawinski P. Markett S. Drewelies J. Düzel S. Demuth I. Steinhagen-Thiessen E. (2022). Linking brain age gap to mental and physical health in the berlin aging study II. *Front. Aging Neurosci.* 14:791222. 10.3389/fnagi.2022.791222 35936763 PMC9355695

[B16] Jeon S. K. Joo I. Park J. Yoo J. (2024). Automated hepatic steatosis assessment on dual-energy CT-derived virtual non-contrast images through fully-automated 3D organ segmentation. *La Radiol. Med.* 129 967–976. 10.1007/s11547-024-01833-8 38869829 PMC11252222

[B17] Jiang R. Wu J. Rosenblatt M. Dai W. Rodriguez R. X. Sui J. (2023). Elevated C-reactive protein mediates the liver-brain axis: A preliminary study. *EBioMedicine* 93:104679. 10.1016/j.ebiom.2023.104679 37356206 PMC10320521

[B18] Jodeiri Farshbaf M. Alvina K. (2021). Multiple roles in neuroprotection for the exercise derived myokine irisin. *Front. Aging Neurosci.* 13:649929. 10.3389/fnagi.2021.649929 33935687 PMC8086837

[B19] Kang S. H. Liu M. Park G. Kim S. Y. Lee H. Matloff W. (2023). Different effects of cardiometabolic syndrome on brain age in relation to gender and ethnicity. *Alzheimer’s Res. Therapy* 15:68. 10.1186/s13195-023-01215-8 36998058 PMC10061789

[B20] Kang Y. Park J. S. Yu K. H. Lee B. C. (2009). A reliability validity, and normative study of the Korean-Montreal Cognitive Assessment (K-MoCA) as an instrument for screening of vascular cognitive impairment (VCI). *Korean J. Clin. Psychol.* 28 549–562. 10.15842/kjcp.2009.28.2.013

[B21] Kim J. A. Shin C. Jung I. Park S. Y. Lee D. Y. Yu J. H. (2025). Impact of muscle quality on muscle strength and physical performance beyond muscle mass or diabetes status. *J. Cachexia Sarcopenia Muscle* 16:e13760. 10.1002/jcsm.13760 40035123 PMC11876848

[B22] Kim M. Hwang I. Choi K. S. Lee J. Ryu M. Park J. H. (2025). Normative modeling reveals age-atypical cortical thickness differences between hepatic steatosis and fibrosis in non-alcoholic fatty Liver disease. *Brain Behav.* 15:e70466. 10.1002/brb3.70466 40195091 PMC11975609

[B23] Kolenic M. Franke K. Hlinka J. Matejka M. Capkova J. Pausova Z. (2018). Obesity, dyslipidemia and brain age in first-episode psychosis. *J. Psychiatr. Res.* 99 151–158. 10.1016/j.jpsychires.2018.02.012 29454222

[B24] Kulshreshtha M. Chandel S. (2025). Mean arterial pressure may be a valuable tool for classifying blood pressure in physically actives: A cross-sectional study among females from North India. *Clin. Epidemiol. Global Health* 31:101912. 10.1016/j.cegh.2025.101912

[B25] Lee H. J. Kuo C. Y. Tsao Y. C. Lee P. L. Chou K. H. Lin C. J. (2025). Brain age gap associations with body composition and metabolic indices in an asian cohort: An MRI-Based study. *Arch. Gerontol. Geriatr.* 133:105830. 10.1016/j.archger.2025.105830 40127523

[B26] Lee J. H. Kim D. Kim H. J. Lee C. H. Yang J. I. Kim W. (2010). Hepatic steatosis index: A simple screening tool reflecting nonalcoholic fatty liver disease. *Dig. Liver Dis.* 42 503–508. 10.1016/j.dld.2009.08.002 19766548

[B27] Liem F. Varoquaux G. Kynast J. Beyer F. Kharabian Masouleh S. Huntenburg J. M. (2017). Predicting brain-age from multimodal imaging data captures cognitive impairment. *Neuroimage* 148 179–188. 10.1016/j.neuroimage.2016.11.005 27890805

[B28] López-Otín C. Blasco M. A. Partridge L. Serrano M. Kroemer G. (2013). The hallmarks of aging. *Cell* 153 1194–1217. 10.1016/j.cell.2013.05.039 23746838 PMC3836174

[B29] MacKinnon D. (2012). *Introduction to statistical mediation analysis.* England: Routledge.

[B30] Matsubara Y. Kiyohara H. Teratani T. Mikami Y. Kanai T. (2022). Organ and brain crosstalk: The liver-brain axis in gastrointestinal, liver, and pancreatic diseases. *Neuropharmacology* 205:108915. 10.1016/j.neuropharm.2021.108915 34919906

[B31] Nachit M. Horsmans Y. Summers R. M. Leclercq I. A. Pickhardt P. J. (2023). CT body composition identifies myosteatosis as key mortality predictor in asymptomatic adults. *Radiology* 307:e222008. 10.1148/radiol.222008 37191484 PMC10315523

[B32] Nguyen H. H. Swain M. G. (2023). Avenues within the gut-liver-brain axis linking chronic liver disease and symptoms. *Front. Neurosci.* 17:1171253. 10.3389/fnins.2023.1171253 37521690 PMC10372440

[B33] Pasco J. A. Sui S. X. West E. C. Anderson K. B. Rufus-Membere P. Tembo M. C. (2022). Fatty liver index and skeletal muscle density. *Calcif. Tissue Int.* 110 649–657. 10.1007/s00223-021-00939-9 35028685 PMC9108103

[B34] Pickhardt P. J. (2022). Value-added opportunistic CT screening: State of the art. *Radiology* 303 241–254. 10.1148/radiol.211561 35289661 PMC9083232

[B35] R Core Team (2013). *R: A language and environment for statistical computing.* Vienna: R Core Team.

[B36] Rao G. Peng X. Li X. An K. He H. Fu X. (2023). Unmasking the enigma of lipid metabolism in metabolic dysfunction-associated steatotic liver disease: From mechanism to the clinic. *Front. Med.* 10:1294267. 10.3389/fmed.2023.1294267 38089874 PMC10711211

[B37] Samuel V. T. Shulman G. I. (2012). Mechanisms for insulin resistance: Common threads and missing links. *Cell* 148 852–871. 10.1016/j.cell.2012.02.017 22385956 PMC3294420

[B38] Samuelsson J. Marseglia A. Wallengren O. Lindberg O. Dartora C. Cedres N. (2025). Association of body composition with neuroimaging biomarkers and cognitive function; a population-based study of 70-year-olds. *EBioMedicine* 112:105555. 10.1016/j.ebiom.2024.105555 39788041 PMC11762906

[B39] Smarandache I. G. Maricutoiu L. P. Ilie M. D. Iancu D. E. Mladenovici V. (2022). Students’ approach to learning: Evidence regarding the importance of the interest-to-effort ratio. *High. Educ. Res. Dev.* 41 546–561. 10.1080/07294360.2020.1865283

[B40] Tantai X. Liu Y. Yeo Y. H. Praktiknjo M. Mauro E. Hamaguchi Y. (2022). Effect of sarcopenia on survival in patients with cirrhosis: A meta-analysis. *J. Hepatol.* 76 588–599. 10.1016/j.jhep.2021.11.006 34785325

[B41] Therkelsen K. E. Pedley A. Speliotes E. K. Massaro J. M. Murabito J. Hoffmann U. (2013). Intramuscular fat and associations with metabolic risk factors in the Framingham Heart Study. *Arterioscler. Thromb Vasc. Biol.* 33 863–870. 10.1161/ATVBAHA.112.301009 23349188 PMC3696991

[B42] VanWagner L. B. Terry J. G. Chow L. S. Alman A. C. Kang H. Ingram K. H. (2017). Nonalcoholic fatty liver disease and measures of early brain health in middle-aged adults: The CARDIA study. *Obesity* 25 642–651. 10.1002/oby.21767 28169509 PMC5323279

[B43] Wang J. Yang R. Miao Y. Zhang X. Paillard-Borg S. Fang Z. (2025). Metabolic dysfunction-associated steatotic liver disease is associated with accelerated brain ageing: A population-based study. *Liver Int.* 45:e70109. 10.1111/liv.70109 40296771 PMC12038381

[B44] Weinstein G. Zelber-Sagi S. Preis S. R. Beiser A. S. DeCarli C. Speliotes E. K. (2018). Association of nonalcoholic fatty liver disease with lower brain volume in healthy middle-aged adults in the framingham study. *JAMA Neurol.* 75 97–104. 10.1001/jamaneurol.2017.3229 29159396 PMC5833484

[B45] Wrigglesworth J. K. V. Smith C. (2021). Systemic drivers of age-related functional decline. *GeroScience* 43 25–38. 10.1007/s11357-020-00275-w

[B46] Yan M. Man S. Sun B. Ma L. Guo L. Huang L. (2023). Gut liver brain axis in diseases: The implications for therapeutic interventions. *Signal Transduction Targeted Therapy* 8:443. 10.1038/s41392-023-01673-4 38057297 PMC10700720

[B47] Yassin L. K. Skrabulyte-Barbulescu J. Alshamsi S. H. Saeed S. Alkuwaiti S. H. Almazrouei S. (2025). The microbiota-gut-brain axis in mental and neurodegenerative disorders: Opportunities for prevention and intervention. *Front. Aging Neurosci.* 17:1667448. 10.3389/fnagi.2025.1667448 41104042 PMC12521449

[B48] Yin Y. Guo Q. Zhou X. Duan Y. Yang Y. Gong S. (2022). Role of brain-gut-muscle axis in human health and energy homeostasis. *Front. Nutr.* 9:947033. 10.3389/fnut.2022.947033 36276808 PMC9582522

[B49] Yu J. H. Kim R. E. Y. Jung J. M. Park S. Y. Lee D. Y. Cho H. J. (2021). Sarcopenia is associated with decreased gray matter volume in the parietal lobe: A longitudinal cohort study. *BMC Geriatr.* 21:622. 10.1186/s12877-021-02581-4 34727885 PMC8565062

[B50] Yuan S. Larsson S. C. (2023). Epidemiology of sarcopenia: Prevalence, risk factors, and consequences. *Metabolism* 144:155533. 10.1016/j.metabol.2023.155533 36907247

[B51] Zhao X. Jin L. Sun S. B. (2021). The bidirectional association between physical and cognitive function among chinese older adults: A mediation analysis. *Int. J. Aging Hum. Dev.* 92 240–263. 10.1177/0091415020940214 32677441

[B52] Zhao X. Lynch J. G. Jr. Chen Q. (2010). Reconsidering baron and Kenny: Myths and truths about mediation analysis. *J. Consumer Res.* 37 197–206. 10.1086/651257

