# Supplementary material for: Mediating role of muscle quality in the liver–brain axis: integrated analysis of CT markers of body composition, brain aging, and biomarkers
Source: Front Aging Neurosci. 2025 Nov 17;17:1676721. doi: 10.3389/fnagi.2025.1676721 (PMC12665701; doi:10.3389/fnagi.2025.1676721)
Supplement: Supplementary file 1 [file Data_Sheet_1.docx]

Supplementary material

**Figure S1.** Mediation analysis results for the direct, indirect, and total effects, with brain age gap (BAG) as the independent variable, muscle density (HU) as the mediator, and the liver attenuation index (LAI) as the dependent variable.


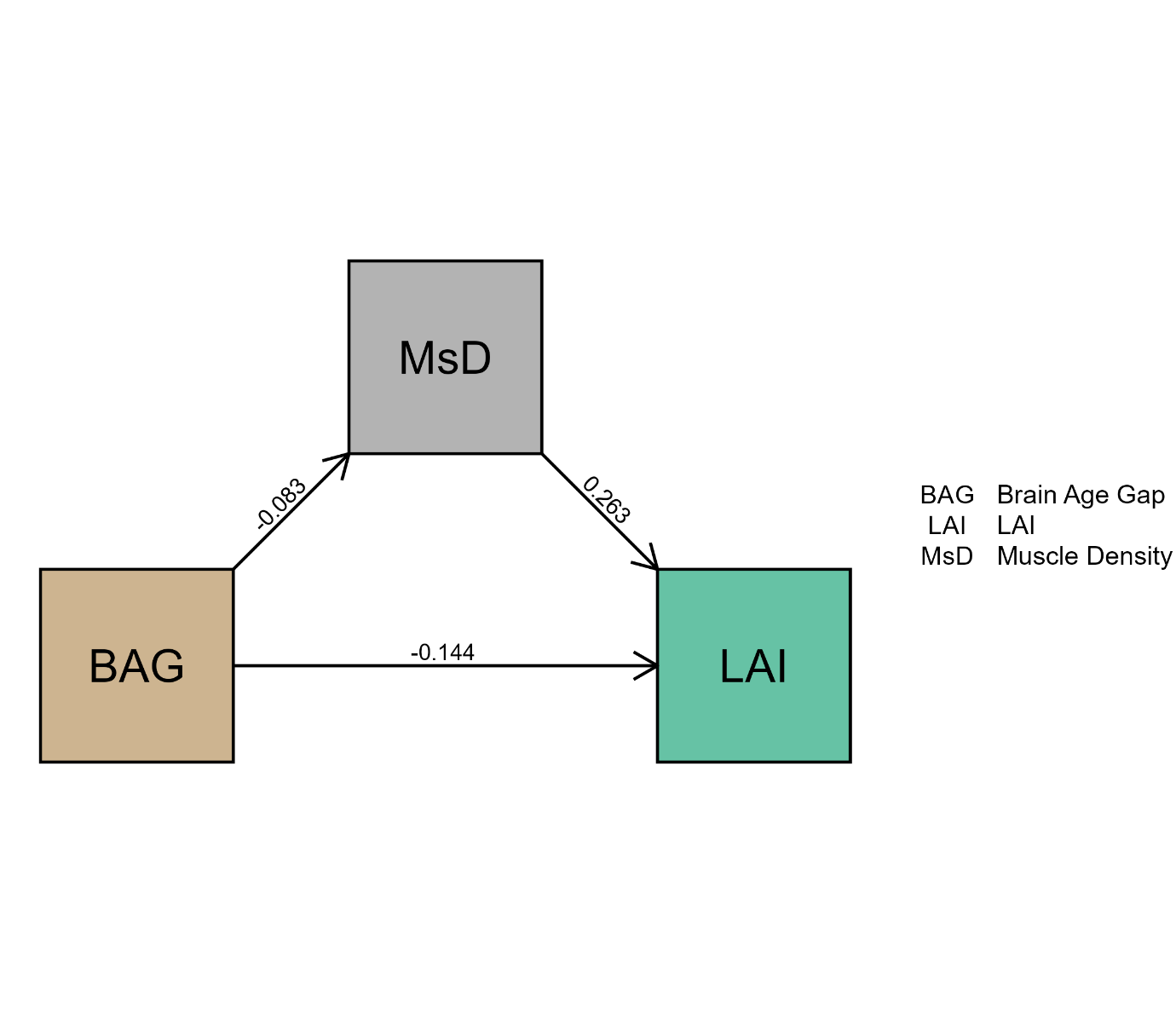


| **Effect type** | **Pathway** | **Effect (B)** | **SE** | **z** | **p** | **95% CI (lower)** | **95% CI (upper)** |
| --- | --- | --- | --- | --- | --- | --- | --- |
| **Direct effect** | BAG → LAI (c') | −0.144 | 0.0046 | −3.141 | 0.002 | −0.240 | −0.055 |
| **Indirect effect** | LAI → Muscle density → BAG (a × b) | −0.022 | 0.0006 | -3.390 | <0.001 | −0.0035 | −0.0010 |
| **Total effect** | LAI → BAG (c) | −0.166 | 0.0046 | −3.606 | <0.001 | −0.258 | −0.076 |

LAI, Liver attenuation index; BAG, brain age gap; SE, standard error; CI, confidence interval.

**Figure S2.** Statistical path plot of liver attenuation index as a predictor of accelerated brain age, mediated by normalized muscle volume. Note that the positive relation between muscle volume and the brain age gap, that is opposite to the model with muscle density as the mediator.


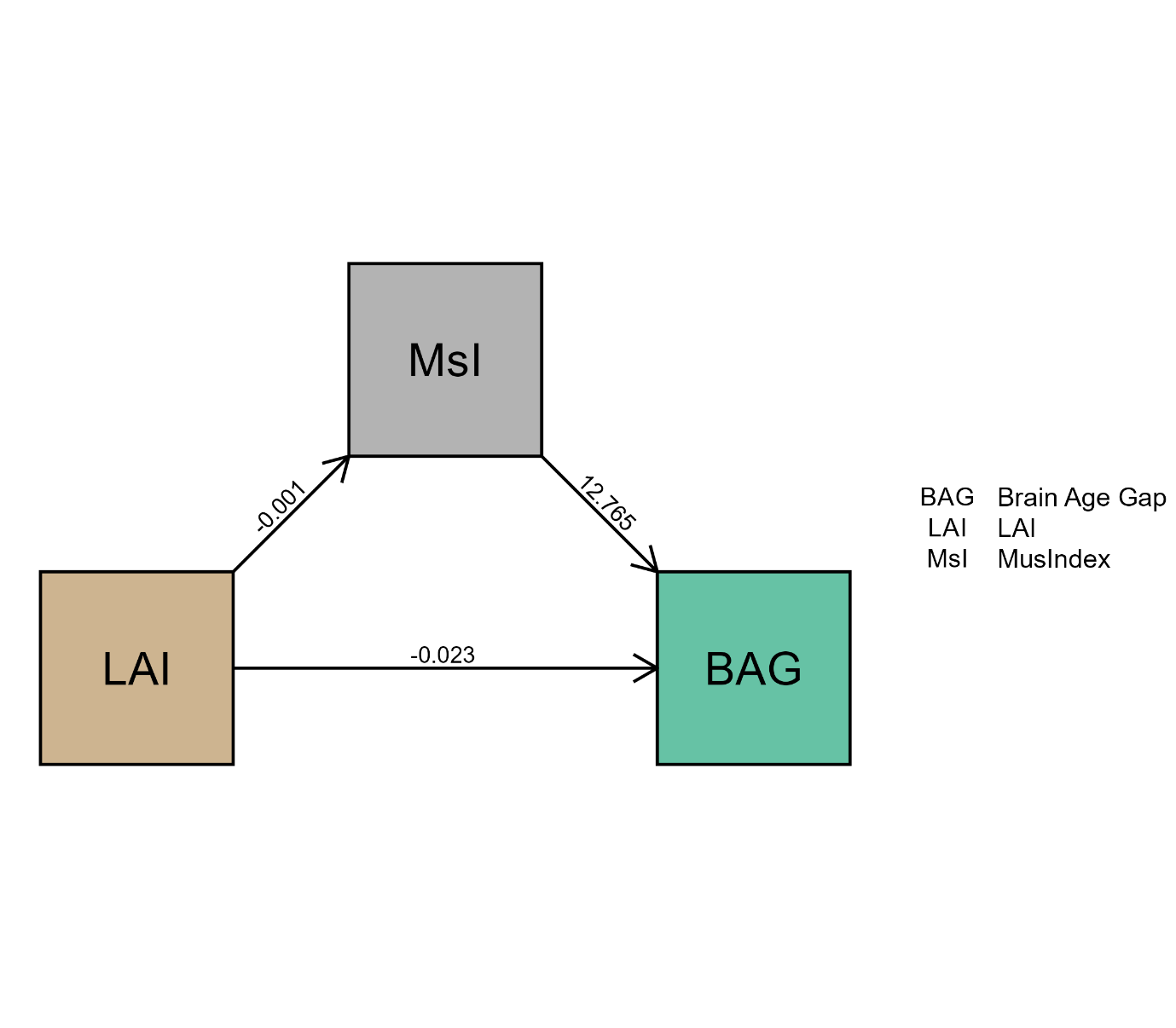


**Figure S3.** The edge stability estimates for networks using bootstrapping. he black line indicates the bootstrapped mean values of the confidence interval, and the gray area the 95% confidence intervals. For both networks, the edge strength between muscle density and brain age gap excludes zero.


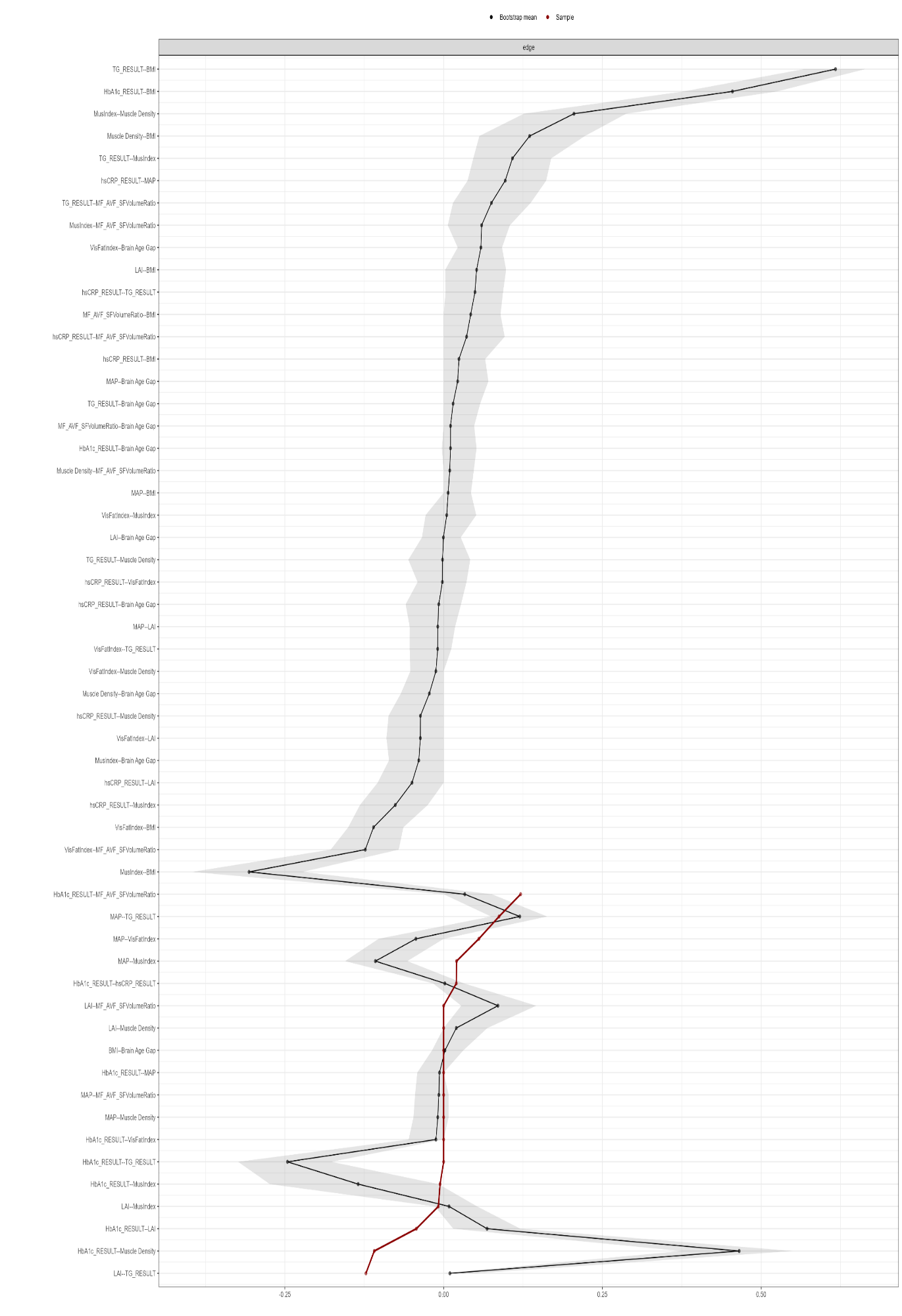

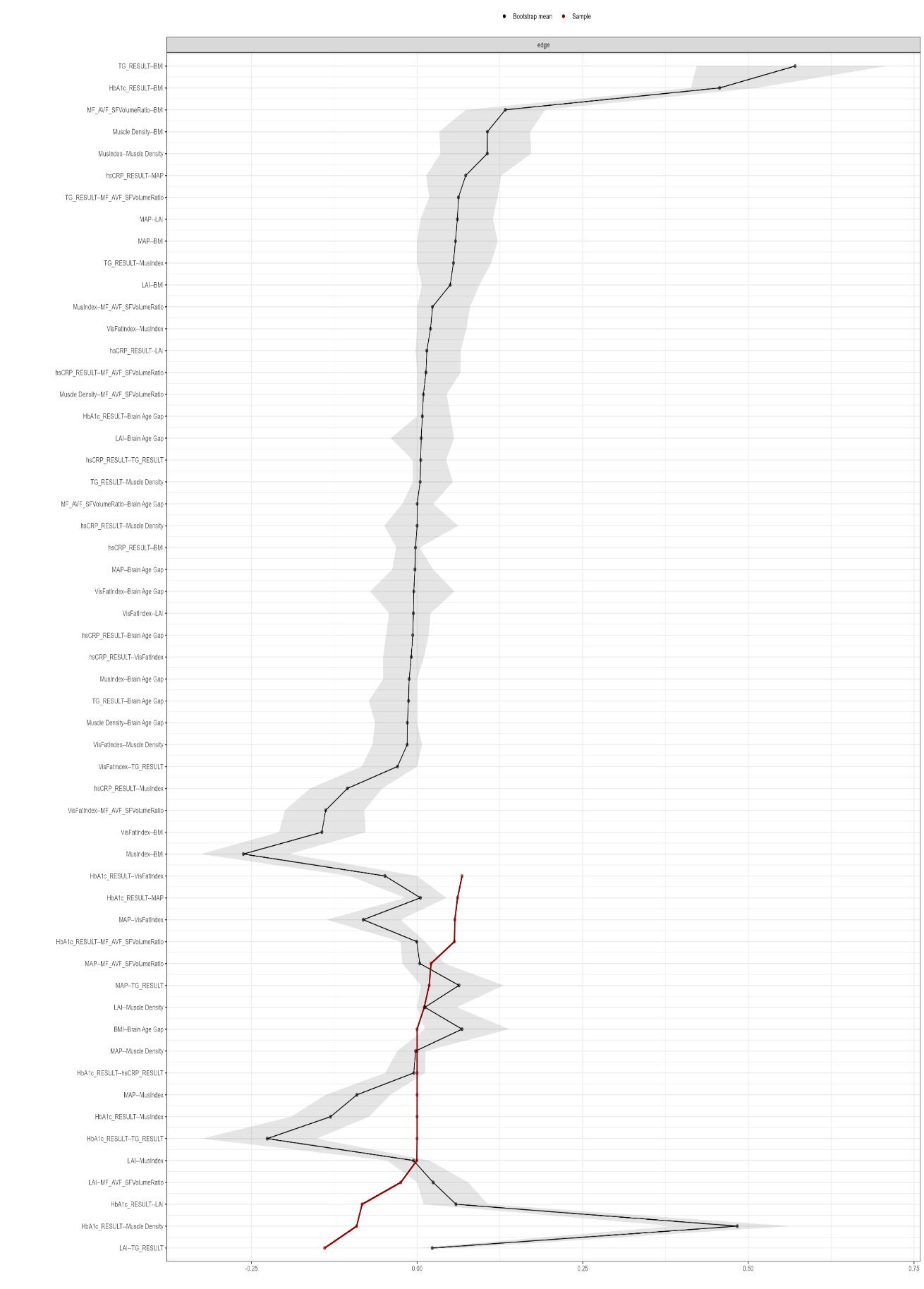


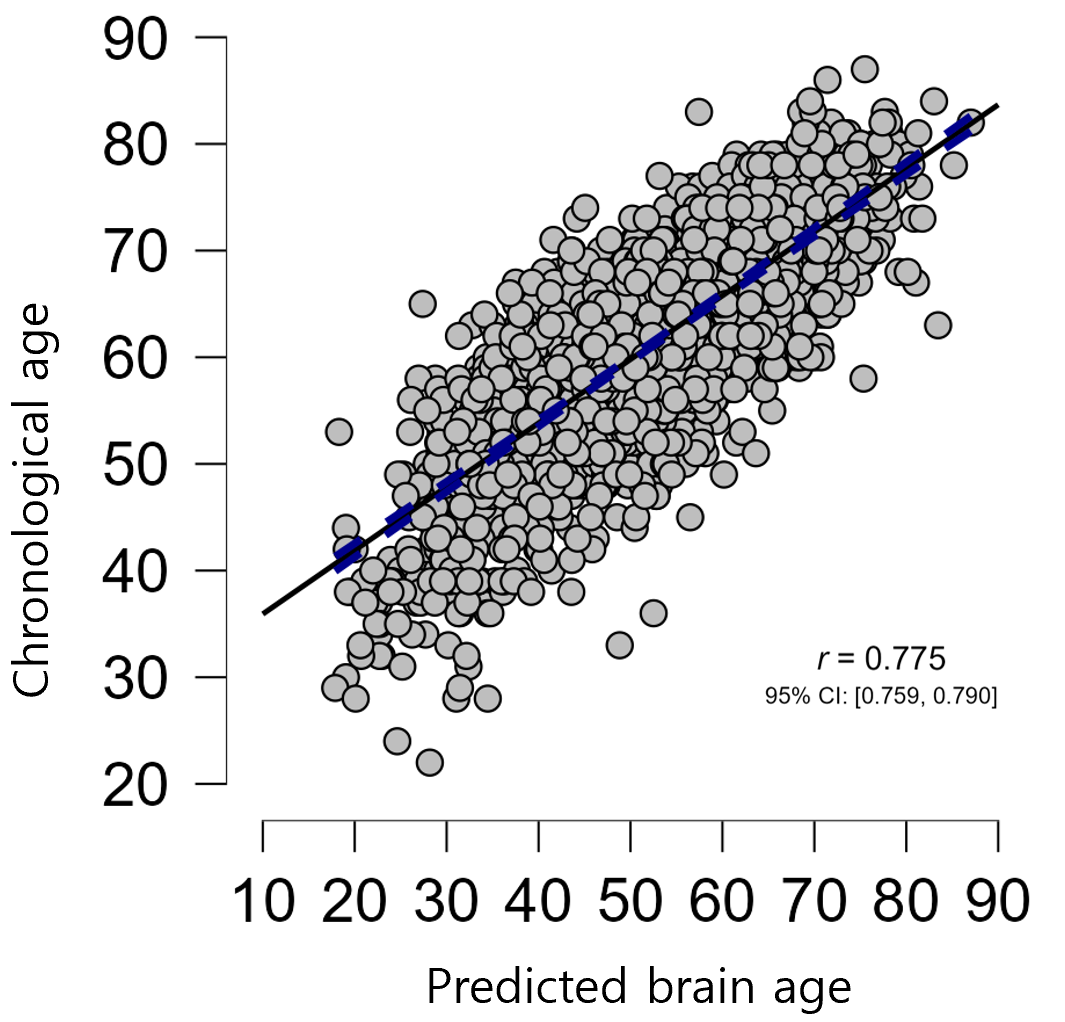


**Figure S4.** Scatter plot between the chronological age and predicted brain age.
